# *Clonorchis sinensis* and *Echinostoma hortense* detected by endoscopy and molecular characterization: two case reports and update on diagnosis

**DOI:** 10.3389/fmed.2024.1515539

**Published:** 2025-01-22

**Authors:** Lijia Wen, Benhe Wang, Hui Zhang

**Affiliations:** ^1^Department of Hepatobiliary and Pancreatic Surgery, General Surgery Center, The First Hospital of Jilin University, Changchun, China; ^2^Department of Parasitic Diseases, Jilin Provincial Center for Disease Control and Prevention (Jilin Provincial Academy of Preventive Medicine), Changchun, China; ^3^Department of Laboratory Medicine, The First Hospital of Jilin University, Changchun, China

**Keywords:** *Clonorchis sinensis*, *Echinostoma hortense*, trematode, diagnosis, endoscopy, molecular characterization, ITS, COX-1

## Abstract

*Clonorchis sinensis* (*C. sinensis*) and *Echinostoma hortense* (*E. Hortense*) infections represent significant food-borne zoonotic parasitic diseases. *C. sinensis* stands as the primary parasite underlying cholangitis, cholelithiasis, and even cholangiocarcinoma, whereas *E. Hortense* parasitizes the duodenum. Unfortunately, the non-specific clinical presentations of these two trematodes frequently mislead clinicians, resulting in overlooks or misdiagnoses, and consequently, inadequate treatment. We diagnosed digestive system trematodes through endoscopic observation and molecular methods. Herein, we present a case where *C. sinensis* was definitively diagnosed through direct observation under duodenoscopy. For the first time, we captured the entire migratory process of the parasite from the common bile duct into the intestinal lumen. In another case, multiple active trematodes were detected on the duodenal wall under duodenoscopy, subsequently extracted using endoscopic forceps. Based on the morphology of the worms and their eggs, an initial diagnosis of *Echinostoma* was made. To confirm the species, we designed primers targeting the ribosomal ITS (internal transcribed spacer) and mitochondrial COX-1 (cyclooxygenase-1) genes, followed by PCR amplification and sequencing. The results conclusively matched the sequence of *E. Hortense*, verifying our final diagnosis. Our proposed approach, integrating endoscopy with molecular characteristics, offers novel strategies for diagnosing trematode infections. This methodology represents a significant advancement in the field, enhancing the accuracy and timeliness of treatment interventions.

## 1 Introduction

*Clonorchis sinensis* (*C. sinensis*), commonly known as liver fluke, is a parasite that poses a significant health threat (1). *C. sinensis* is prevalent mainly in East Asian countries, including Japan, Korea, Vietnam and China, especially in certain geographical regions, approximately over 750 million people worldwide are at risk of liver fluke infection. Humans or other mammals are infected by consuming raw or undercooked freshwater fish containing metacercaria (2). The fluke’s infection can lead to serious health issues, including an increased risk of bile duct stones and cholangiocarcinoma (3, 4). Unfortunately, diagnosis of *C. sinensis* infection is often challenging, resulting in a high rate of missed diagnoses (5). Currently, the primary diagnostic method relies on microscopic examination of stool samples, which requires skilled personnel.

*Echinostoma hortense* (*E. hortense*) belongs to the class of Trematoda and genus of *Echinostoma* (6). Humans are mostly infected by eating fish, frogs and snails containing metacercariae. Loach has been proved to be the second intermediate host of *E. hortense* (7). *E. hortense* are similar to *C. sinensis* in that they are mainly distributed in East Asian countries and are mostly reported in the form of case reports, with the highest number of reports coming from South Korea (8). Most of patients have no obvious symptoms, so it is difficult to diagnose and delay treatment.

*C. sinensis* and *E. hortense* are two different types of trematodes, but they have some commonalities (9). First, they belong to the flatworm phylum and both possess a sucker structure, typically comprising a mouth sucker and a ventral sucker, for attaching to and extracting nutrients from the host. Second, they share similar life cycles and parasitism. Both of them belong to indirect developmental trematodes, with complex life histories that require multiple stages and intermediate hosts. Their infectious stage is usually metacercaria, which are transmitted to the final host such as humans and mammals. Last, both of them can parasitize the digestive tract of the host and cause a series of clinical symptoms, such as diarrhea, abdominal pain, and dyspepsia.

## 2 Methods

The data in the medical records were collected as part of routine diagnosis and treatment, sharing data of the descriptive retrospective study anonymously was agreed by patients.

## 3 Results

### 3.1 Case 1

**Figure 1:**
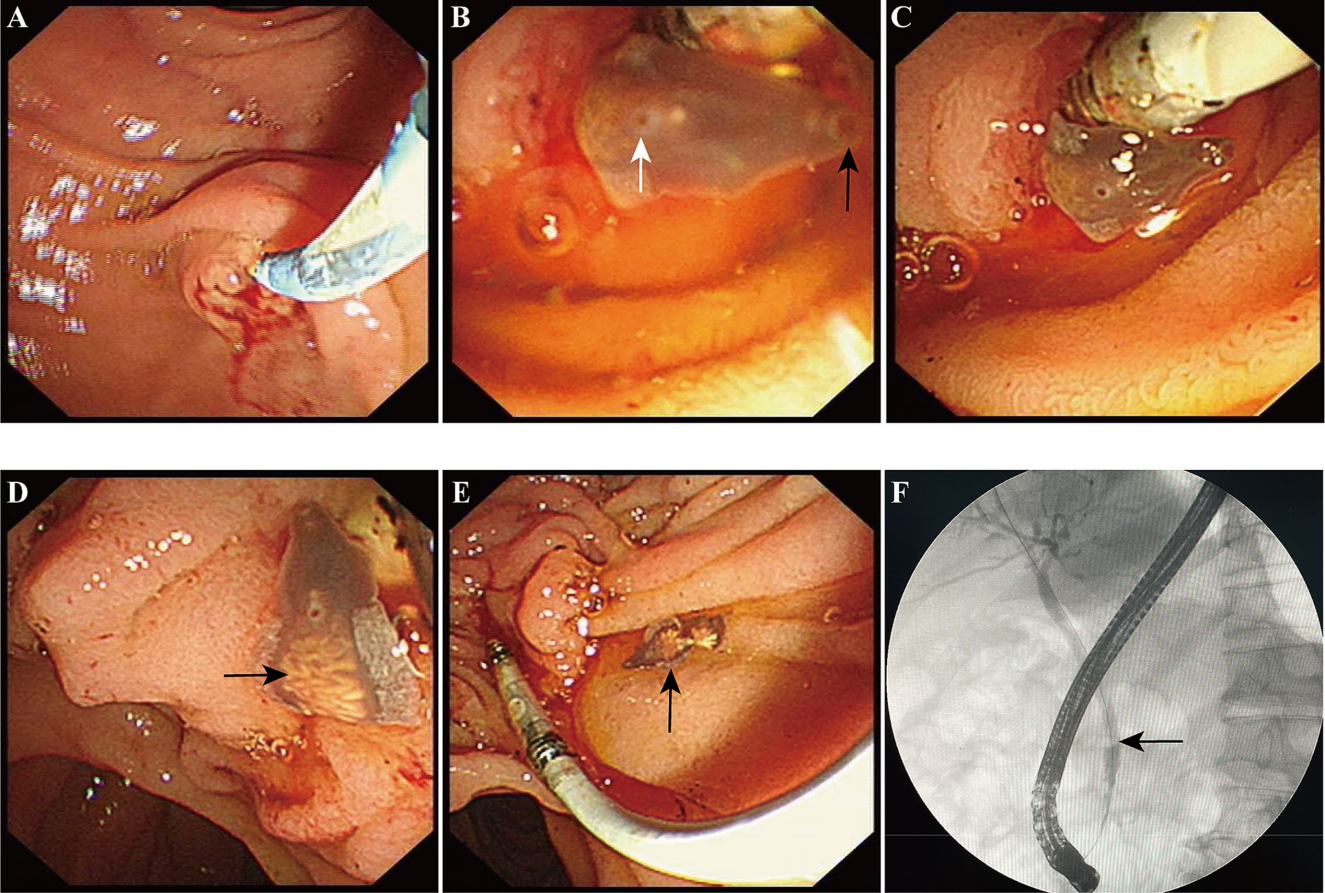
The diagnosis and migration of *Clonorchis sinensis* under direct endoscopic observation. **(A)** The conventional procedure of common bile duct intubation and nipple incision. **(B)** The anterior portion of *C. sinensis* is clearly visible as it emerges from the Vater’s ampulla. Black arrow pointed to the oral sucker, white arrow denoted the abdominal sucker. **(C,D)** The anterior and most of the body of *C. sinensis* have already entered the duodenal lumen, black arrow indicates the uterus of *C. sinensis*. **(E)** The worm had completely entered the duodenal cavity. **(F)** Cholangiography revealed the presence of a common bile duct stone shadow.

**Figure 2:**
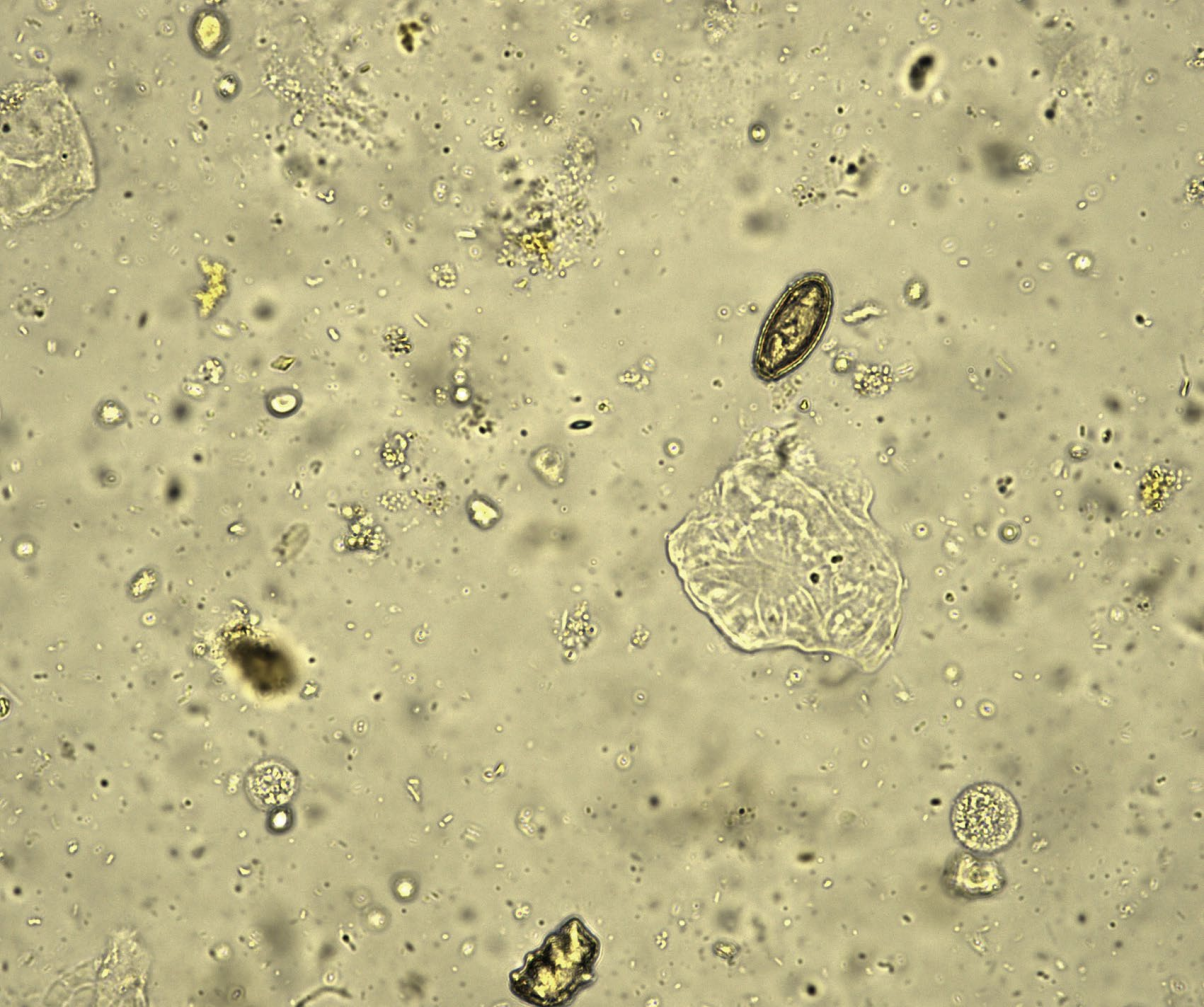
Multiple repeated fecal examinations finally identified the presence of *C. sinensis* eggs.

A 54-year-old male patient presented with abdominal pain, prompting his admission to the hospital. Upon admission, blood biochemistry tests revealed elevated levels of total bilirubin at 37 μmol/L (normal range: 0–26 μmol/L), direct bilirubin at 11.6 μmol/L (normal range: 0–6.8 μmol/L), aspartate aminotransferase (AST) at 59.5 IU/L (normal range: 15–40 IU/L), alanine aminotransferase (ALT) at 304 U/L (normal range: 9–50 U/L), *γ*-glutamyltranspeptidase at 465 U/L (normal range: 10–60 U/L), and alkaline phosphatase (ALP) at 457 U/L (normal range: 40–129 U/L).The patient lived in the endemic area of *C. sinensis* and had the habit of eating freshwater fish for many times, but no parasitic eggs were detected in the patient’s stool upon admission. An abdominal computed tomography (CT) scan suggested the presence of stones at the terminal bile duct, leading to the decision to perform endoscopic retrograde cholangiopancreatography (ERCP)-guided bile duct lithotomy. During the procedure, following routine duodenal papilla intubation and sphincterotomy (Figure 1A), an unexpected discovery was made: worms emerging from the ampulla of Vater (Figure 1B). These worms exhibited a narrow, elongated, and twisted appearance, coupled with remarkable extensibility. The body exhibits a flat form, devoid of spiny epidermis, and the diameter of the oral sucker surpasses that of the ventral sucker (Figure 1C). Upon the worm’s emergence, the intricate branching of the ovaries became distinctly visible, as shown in Figure 1D. Ultimately, the worm fully navigated into the intestinal cavity (Figure 1E). Cholangiography analysis revealed a void in filling at the terminus of the common bile duct, indicative of a stone shadow (Figure 1F). Based on meticulous morphological assessment of the parasite’s body and repeated fecal egg examinations (Figure 2), a diagnosis of *C. sinensis* infection was confirmed. Subsequently, the patient was diagnosed with bile duct stones concurrently infected with *C. sinensis*. Following the removal of the stones, the patient underwent a three-day treatment with praziquantel at a dose of 210 mg/Kg body weight, resulting in a complete resolution of abdominal pain symptoms.

### 3.2 Case 2

**Figure 3:**
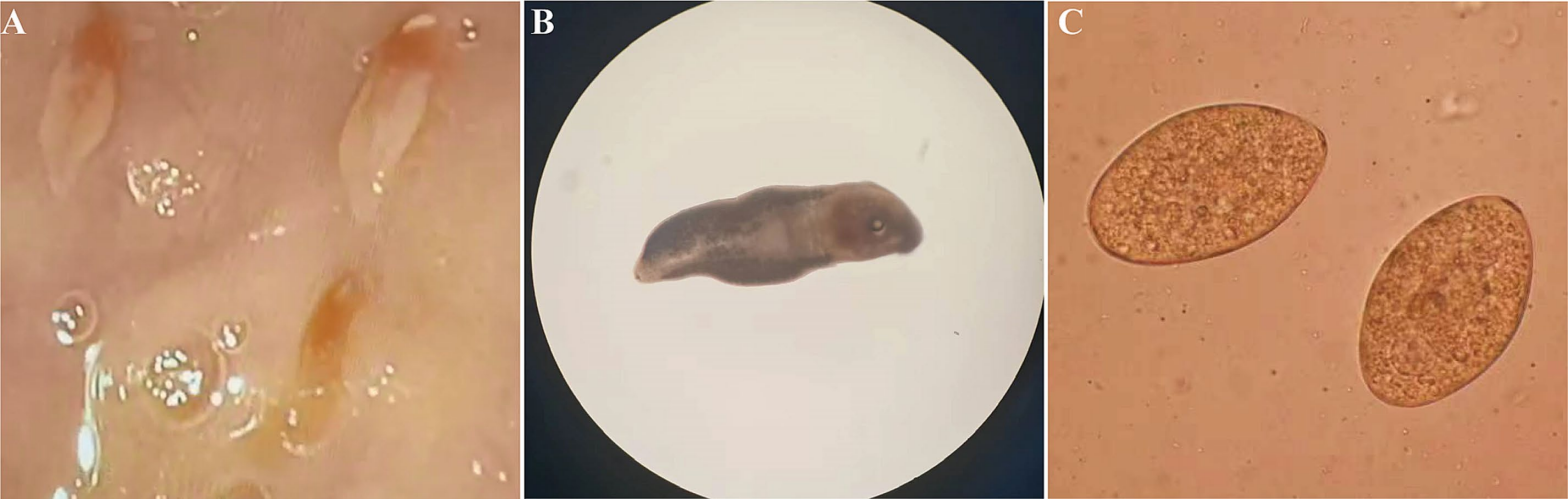
Morphological characterization of *Echinostoma* worms and their Eggs. **(A)** Gastro-duodenoscopic visualization of worms adhered to intestinal mucosa. **(B)** Microscopic examination of worm morphology *in vitro*. **(C)** Egg morphology obtained from worms.

A 77-year-old female patient was admitted to a local hospital due to persistent upper abdominal pain and intermittent nausea that had persisted for 15 days. Laboratory indicators cannot be acquired. During a gastroduodenoscopy procedure, a significant finding emerged: multiple motile trematodes were observed adhering to the duodenal wall (Figure 3A). These worms exhibited a distinct, elongated leaf-like shape, approximately 8 mm in length, featuring red markings at their anterior ends. Their suckers were firmly entrenched on the intestinal wall. Two of these parasites were successfully extracted from the duodenum using endoscopic forceps. A detailed examination under a stereomicroscope revealed an opaque worm body, accompanied by prominently visible oral and abdominal suckers (Figure 3B). Concurrently, exhaustive microscopic analyses of the patient’s stool samples failed to detect any parasite eggs. Then eggs were obtained from the adult worms, their morphological characteristics mirrored those of *Echinostoma* (Figure 3C).

**Figure 4:**
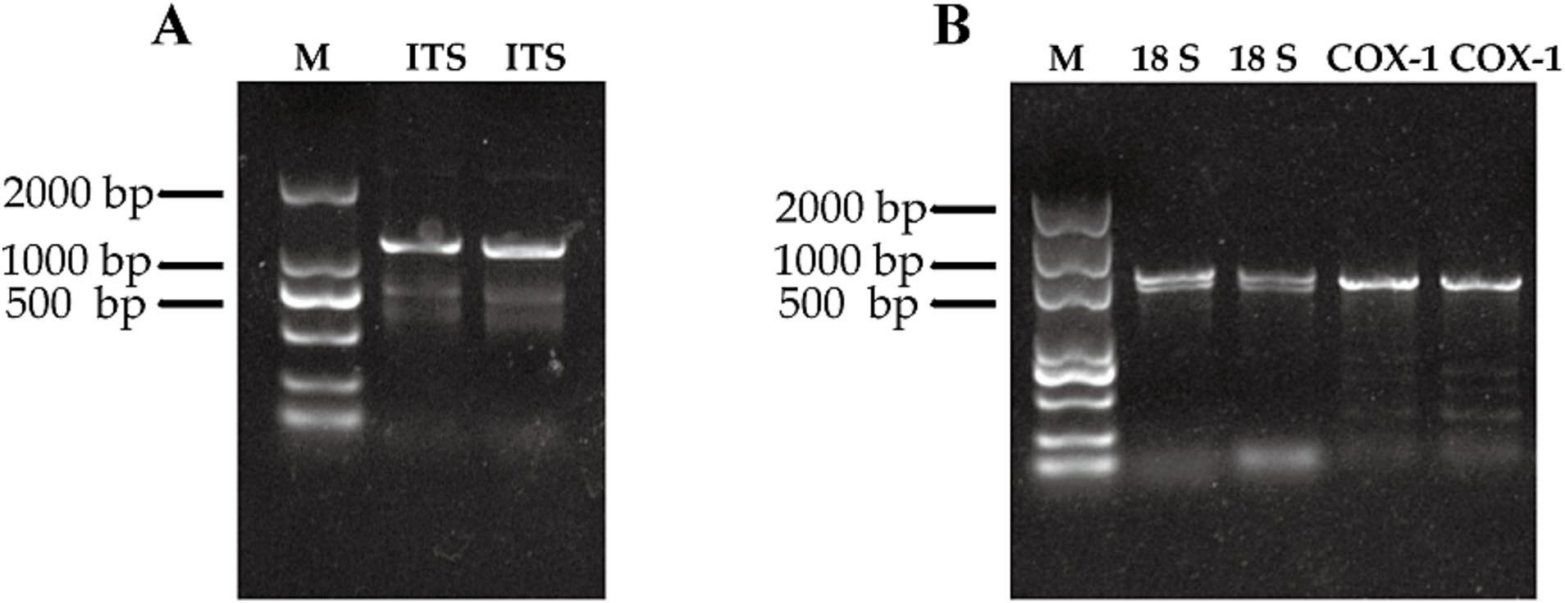
Image of nucleic acid gel after gene amplification by PCR. **(A)** Amplification results of ITS sequences of two *E. hortense* worms. **(B)** Amplification results of 18S and COX-1 sequences of two *E. hortense* worms.

To definitively identify the species, the worms’ DNA was extracted. The amplification conditions for PCR are as follows: pre-denaturation at 94°C for 5 min; enter the cycle: denaturation at 98°C for 30 s, annealing at 55°C for 30 s, extension at 72°C for 60 s, for a total of 35 cycles. The ribosomal internal transcribed spacer (ITS) sequence was amplified via PCR. The ITS forward primer sequence was 5′ -TTA GTT TCT TTT CCT CCG CT-3′, and the reverse primer sequence was 5′ -GTA GGT GAA CCT GCG GAA GGA TCA TT-3′. The gel image distinctly reveals a clear band at 1200 bp, corroborating the expected size of the target gene (Figure 4A). Then this target band was excised, purified, and subsequently sequenced. Homology analysis of the obtained sequence indicated a remarkable similarity with *Echinostoma hortense* and *Isthmiophora hortense*, achieving 98 and 93% sequence homology, respectively. To further substantiate our findings, we designed specific primers for the mitochondrial cyclooxygenase-1 (COX-1) gene and 18S ribosomal RNA gene. COX-1 forward primer: TAA GTC CTG TCG CTG CTA, and COX-1 reverse primer: CCT CCA CCA ACC TAA CCT. 18S forward primer: 5’-CTG GTT GAT CCT GCC AGT AGT C-3′, 18S reverse primer: 5’-ACG ACT TTT ACT TCC TCT AAA T-3′. The PCR product of 18S was sequenced, and the results showed that the product had two peaks, proving that the product is non-specific. The COX-1 sequences were successfully amplified (Figure 4B), and homology analysis unveiled a perfect match of 100% with the COX-1 gene of *Echinostoma hortense*, conclusively diagnosing the patient’s condition. Upon delving into the patient’s medical history, it emerged that she had consumed raw loach approximately 3 months prior to the onset of her symptoms. Following a three-day course of praziquantel therapy at a dose of 100 mg/Kg body weight, the abdominal discomfort notably subsided.

## 4 Discussion

Two studies that diagnose *C. sinensis* under direct endoscopy are presented, utilizing laparoscopic exploration and peroral cholangioscopy respectively, for both diagnosis and treatment purposes (10, 11). Liu et al. (10) reported 2 cases with pain and jaundice, who ate raw fish, found cholangiolithiasis and cholecystolithiasis. During laparoscopic cholecystectomy, adult worms of *C. sinensis* were found in the intrahepatic bile ducts, and a description of their morphology was provided. For another case (11), doctors conducted an endoscopic ultrasonography and ERCP-guided peroral cholangioscopy on an 80-year-old patient with biliary dilation. *C. sinensis* was found in both the common bile duct and intrahepatic bile ducts, and the worms were captured and removed using a basket.

The utilization of endoscopic direct observation for diagnosing *C. sinensis* infection boasts several pivotal advantages. Foremost among them is the unparalleled capacity to visually discern the parasite, thereby furnishing a conclusive and unambiguous diagnosis that significantly mitigates the likelihood of misdiagnosis or false negatives. Furthermore, this method exhibits high sensitivity, ensuring an accurate and reliable assessment of the infection status. Lastly, endoscopy presents a unique opportunity for a combined assessment, facilitating a simultaneous examination of various gastrointestinal pathologies and thereby enabling a comprehensive evaluation of the patient’s overall condition.

However, relying solely on morphological methods for the accurate diagnosis of *Echinostoma* poses a significant challenge, given the vast diversity of parasites encompassing over 600 species within 11 subfamilies and 51 genera (12–14). The precise identification of these parasites forms the cornerstone for the effective prevention, control, and scientific research pertaining to parasitic diseases. We summarized the cases diagnosed with *E. hortense* and published in English in the past and found that most of them relied solely on morphological diagnosis, which is not reliable for distinguishing a wide variety of *Echinostoma* (Table 1).

**Table 1:** Human cases infected with *Echinostoma hortense.*

Case no.	Author	Country	Year	Age (Years) /Sex	Symptoms	Laboratory findings	Site of infection	Diagnostic methods
1	Seo et.al. (21)	Korean	1983	21/M	No symptoms	–	–	Fecal examination
2	Chai et.al. (22)	Korean	1994	55/M	Abdominal pain	animia	Duodenum	Gastrointestinal endoscopy
3	Ryang et.al. (23)	Korean	1985	38/M	Abdominal pain	–	–	Fecal examination
4	Ryang et.al. (23)	Korean	1985	20/M	Abdominal pain and diarrhea	–	–	Fecal examination
5	Cho et.al. (24)	Korean	2003	81/M	Epigastric discomfort	Abnormality	Duodenum	Gastrointestinal endoscopy
6	Chang et.al. (25)	Korean	2005	55/F	Abdominal pain	–	Duodenum	Gastroduodenal endoscopy
7	Tanaka et.al. (26)	Japan	2002	82/F	Abdominal discomfort	Elevation of CEA	Duodenum	Gastrointestinal endoscopy

Specifically, we employ two distinct and conserved sequences, ITS and COX-1, as invaluable tools for species identification. ITS, encompassing the internal transcribed spacer regions (ITS1, 5.8S rRNA, and ITS2), is situated between the large and small subunit rRNA genes within the ribosomal DNA (rDNA) (15). Its unique blend of high variability within species and marked differences between species, coupled with a sequence length typically ranging from 1,000 to 1,200 base pairs (bp), renders it exception-ally suitable for species-level identification and phylogenetic analyses (16). Notably, the ITS evolution rate surpasses that of 18S rDNA by tenfold, further underscoring its utility in these endeavors. By sequencing ITS and comparing it with established *Echinostoma* ITS sequences, we achieve accuracy in taxonomic classification, resolving controversies and elucidating both intraspecific and interspecific relationships. Additionally, we harness the power of COX-1, a highly conserved yet rapidly evolving and maternally inherited gene prevalent in various organisms, including parasites (17, 18). This gene, despite its length variability, is commonly analyzed in partial sequences due to its unique combination of conservatism and evolutionary dynamics. In our study, through PCR amplification and sequencing of both ITS and COX-1, we conclusively authenticated the species as *E. hortense*, demonstrating the efficacy of our approach in advancing *Echinostoma* taxonomy.

From a One Health perspective (19), these clinical findings are invaluable for several reasons: First, identifying infection sources. The diagnosis highlights the need to identify and manage the sources of infection. In the context of *C. sinensis* and *E. hortense*, this typically involves tracing the contamination of freshwater fish, which serve as the intermediate hosts for this parasite. By exploring the infection sources, public health authorities can implement control measures to prevent the spread of the parasite to humans through contaminated food. Second, assessing food habits and risk. The clinical cases also draw attention to the risks associated with specific food habits, particularly the consumption of raw or undercooked freshwater fish. From a One Health standpoint, understanding these food habits is crucial for developing targeted interventions to change behaviors that increase the risk of infection. This may involve educational campaigns, regulatory measures, or both, to inform the public about the risks and promote safe food practices. Last, promoting interdisciplinary collaboration. The One Health approach encourages collaboration between medical professionals, veterinarians, environmental scientists, and other stakeholders to address complex health issues. This collaboration can lead to a more comprehensive understanding of the parasite’s lifecycle, transmission routes, and impact on human and animal health. This, in turn, can facilitate the development of integrated control strategies that address the parasite at multiple levels (20).

## 5 Conclusion

Traditional diagnosis of trematode infections heavily reliant on fecal microscopy, suffering from low sensitivity and a high incidence of missed diagnoses. As a complementary approach, endoscopy with PCR-based molecular diagnosis offering a more comprehensive and reliable method. This provides more accurate methods for the prevention, diagnosis, and treatment of diseases, and offers methodological support for the One Health system.

## Data Availability

The original contributions presented in the study are included in the article/supplementary material, further inquiries can be directed to the corresponding author.
